# Antibiotic postprescribing modification opportunities among nursing home residents treated for urinary tract infection

**DOI:** 10.1017/ice.2022.202

**Published:** 2023-06

**Authors:** Madeline C. Langenstroer, Sally Jolles, Tamanna Hossin, Anna Nora, Mozhdeh Bahrainian, Christopher Crnich, Lindsay Taylor

**Affiliations:** 1 Department of Population Health, University of Wisconsin School of Medicine and Public Health, Madison, Wisconsin; 2 Department of Medicine, University of Wisconsin School of Medicine and Public Health, Madison, Wisconsin; 3 Department of Emergency Medicine, Yale New Haven Hospital, New Haven, Connecticut; 4 William S. Middleton VA Hospital, Madison, Wisconsin; 5 Department of Ophthalmology and Visual Sciences, University of Wisconsin School of Medicine and Public Health, Madison, Wisconsin

## Abstract

**Objective:**

To characterize opportunities to postprescriptively modify antibiotic prescriptions initiated for treatment of suspected urinary tract infection (UTI) in nursing homes.

**Design:**

Cross-sectional cohort study.

**Methods:**

Data from the health records of residents treated for UTI between 2013 and 2014 in 5 Wisconsin nursing homes were abstracted using a structured approach. Explicit definitions were used to identify whether the prescribed antibiotic could have been stopped, shortened, or changed to a nonfluoroquinolone alternative. Antibiotic treatments appropriately modified by study nursing home providers in real time were not considered modifiable. Identification of >1 potential modification opportunity (eg, stop and shorten) per antibiotic treatment event was permitted.

**Results:**

In total, 356 eligible antibiotic treatment courses among 249 unique residents were identified. Only 59 antibiotic courses prescribed for treatment of suspected UTI (16.6%) were not amenable to any modification. Discontinuation of treatment due to lack of signs or symptoms of infection was the most frequently identified potential modification opportunity (66.2%). Although less common, substantial numbers of antibiotic treatment courses were potentially amenable to shortening (34%) or agent change (19%) modifications. If applied in concert at 72 hours after antibiotic initiation, stop and shorten modifications could eradicate up to 1,326 avoidable antibiotic days, and change modifications could remove a 32 remaining avoidable fluoroquinolone days.

**Conclusions:**

Substantial opportunity exists to enhance the quality of antibiotic prescribing for treatment of suspected UTI in nursing homes through postprescriptive review interventions. Additional studies examining how to best design and implement postprescriptive review interventions in nursing homes are needed.

Antibiotic overuse and misuse are common problems in nursing homes. Between 8% and 11% of the 1.4 million individuals who reside in nursing homes are treated with an antibiotic each day.^1^ A resident living in a nursing home for at least 6 months has a >50% risk of being prescribed at least 1 antibiotic course.^2^ Urinary tract infection (UTI) is the most frequent indication for antibiotics in nursing homes, and fluoroquinolones are the most commonly prescribed antibiotic class for UTIs.^3^ Antibiotic treatment is associated with several possible harms, such as adverse drug reactions, *Clostridioides difficile* infection (CDI), and risk of colonization with and spread of multidrug-resistant organisms.^4^ Fluoroquinolone use is associated with additional risk of tendinopathy, confusion, and QT prolongation.^5^ Many of these harmful events are potentially avoidable; it has been estimated that 25%–75% of antibiotic use in nursing homes is inappropriate.^6,7^

Adoption of programs and interventions aimed at improving the quality of antibiotic prescribing is increasingly common in nursing homes.^8^ Many of the efforts to improve antibiotic prescribing practices in nursing homes employ preprescriptive interventions focused on avoiding initiation of unnecessary treatments.^9^ The use and effects of postprescriptive interventions, such as prospective audit and feedback as well as antibiotic timeouts, have been less well studied in this setting.^10–12^ Achieving a better understanding of how much opportunity there is to modify antibiotics after their initiation is needed to support the development and successful implementation of effective postprescriptive stewardship interventions in the nursing home setting.

The objective of this study was to characterize the frequency and types of potential postprescriptive modifications of antibiotics initiated for treatment of suspected UTI in nursing homes, specifically opportunities to stop, shorten, and/or change to nonfluoroquinolone alternatives.

## Methods

Data for this study were collected during a retrospective cross-sectional chart review study conducted in 5 Wisconsin nursing homes between January 2013 and September 2014. Participating nursing homes were purposively sampled for their geographic proximity to the University of Wisconsin–Madison. Trained research staff collected and managed study data using REDCap electronic capture tools hosted by the Department of Medicine at the University of Wisconsin School of Medicine and Public Health. Resident identifiers were not recorded as part of this study, and the Human Subjects Institutional Review Board at the University of Wisconsin School of Medicine and Public Health approved this study protocol.

An antibiotic course was included in the study if it was (1) prescribed for treatment of a UTI, (2) administered systematically, and (3) initiated in the nursing home or following a clinic or emergency department (ED) encounter without an intercurrent hospital admission. Antibiotic treatment courses prescribed for an indication other than UTI or initiated during hospitalization and continued after transfer to the nursing home were excluded from the analysis. Research staff reviewed resident medication administration records, nursing staff and provider records, provider orders, and results of laboratory and imaging studies documented or collected 72 hours before and through the second day of eligible antibiotic treatment courses. Information on the antibiotic agent prescribed, duration of treatment, and culture results or susceptibilities, as well as pertinent patient factors (indwelling urinary catheters, vital signs, symptoms, and exam findings), were recorded. The appropriateness of each antibiotic treatment course was assessed using prevailing explicit criteria.^13,14^

Each antibiotic treatment course was evaluated for 3 potential modification opportunities (PMOs): stop, shorten, or change to a nonfluoroquinolone alternative (described below). Opportunities to change to an active therapy when then initial agent was inactive was not specifically reviewed. Modification opportunities were not considered mutually exclusive, and identification of >1 PMO per antibiotic treatment course was possible.

### Stop PMO

The antibiotic treatment course was considered potentially amenable to discontinuation in the absence of supportive clinical and microbiological findings. A “stop PMO” was identified when McGeer or Loeb clinical criteria were not satisfied or when urine cultures were negative even if Loeb clinical criteria were met. Cases were excluded from review if there was not enough information in the medical record, if the antibiotic was started in error, if the patient was transferred to the hospital prior to completion of therapy, if urine culture data became available within 48 hours of discharge, or if the culture data became available after resident discharge.

### Shorten PMO

Microbiologically active antibiotic duration was calculated based on days of antibiotic treatment that targeted the urinary pathogen(s) recovered from culture. Antibiotic courses were excluded from review if there was not enough information in the medical record, if the antibiotic was started in error, if the patient was transferred to the hospital prior to completion of therapy, if the provider stopped therapy due to negative culture results, or if effective antibiotic duration could not be calculated due to either lack culture data or prescription of an effective antibiotic agent. Antibiotics with an effective antibiotic duration of >7 days were considered amenable to potential shortening.

### Nonfluoroquinolone alternative PMO

Fluoroquinolone antibiotic courses for which urine culture data were available and results had been acquired at least 48 hours before the resident was discharged from the facility were considered for review. Cases were excluded if there was not enough information in the medical record, the antibiotic was started in error, the patient was transferred to the hospital prior to completion of therapy, or if a nonfluoroquinolone antibiotic was prescribed. If the available urine culture data indicated an organism susceptible to at least 1 oral nonfluoroquinolone alternative (ie, nitrofurantoin, trimethoprim-sulfamethoxazole, or first-generation oral cephalosporins), the antibiotic was considered potentially amenable to modification.

The proportions of antibiotic courses amenable to each PMO and days of microbiologically active antibiotic duration were calculated. Comparisons of microbiologically active antibiotic durations between nursing home resident groups were performed using *t* tests. A 2-tailed *P* value of <.05 was considered statistically significant. Data and statistical analyses were conducted in R version 1.3.1073 software (R Foundation for Statistical Computing, Vienna, Austria).

## Results

In total, 1,451 antibiotic treatment courses were identified during the cross-sectional study. Antibiotic treatment courses initiated in the hospital (n = 599), including 185 courses for treatment of UTI, were excluded from further analysis. Antibiotic courses initiated in the nursing home or clinic or ED for reasons other than the treatment of UTI (n = 485) were also excluded. Of the 367 antibiotic courses initiated for treatment of suspected UTI, 11 were excluded from the study due to insufficient data to assess any of the 3 treatment modification opportunities (n = 10) and a treatment course that was started in error (n = 1). The final study sample of 356 antibiotic treatments were prescribed to 249 unique residents, with a mean of 1.43 (SD, 0.98) antibiotic courses per resident. The average age of the residents included in this study was 84.3 (SD, 10.5). Participants were predominately female (n = 176, 70.6%) and one-fifth had an indwelling urinary catheter (n = 51, 20.4%). Moreover, 8 different antibiotic classes were prescribed for initial treatment of suspected UTI in this study (Table 1). Of the 356 eligible treatment courses, 352 were evaluated for a “stop PMO,” 286 were evaluated for a “shorten PMO,” and 117 were evaluated for a nonfluoroquinolone alternative PMO (Table 2).

**Table 1.** Initial Antibiotic Therapy by Group

Antibiotic Group	No. (%)
Fluoroquinolones	127 (35.67)
Sulfonamides	75 (21.07)
Nitrofurantoin	61 (18.25)
Cephalosporins	58 (16.29)
Penicillins with and without β-lactamase inhibitors	26 (7.31)
Tetracyclines	4 (1.12)
Fosfomycin	1 (0.25)
Aminoglycosides	1 (0.29)

**Table 2.** Approach to Analyzing Antibiotic Treatment Courses for Postprescribing Modification Opportunities (PMOs)

PMO	Applied Review Criteria	Exclusion Criteria	No. of Antibiotic Courses Reviewed
Stop	Event does not fulfill either: McGeer criteria (clinical + microbiologic criteria) OR Loeb criteria with a positive urine culture	• Discharged from facility before or within 48 h of release of urine culture results (n=4)	352
Shorten	Effective antibiotic duration ≥7 d	Lack of culture data (n=38) Initial UTI diagnosis rejected by provider (n=21) Ineffective therapy (n=4) Discharged prior to end of therapy (n=7)	286
Non-FQ alternative	FQ antibiotics prescribed with the indication of cystitis AND Non-FQ alternative appropriate for urinary pathogen	Non-FQ antibiotic prescribed (n=211) Lack of urine culture data (n=25) Ineffective therapy (n=4) Discharged from facility before or within 48 hours of release of urine culture results (n=3)	117

Note. FQ, fluoroquinolone.

### Stop PMO

**Fig. 1.**
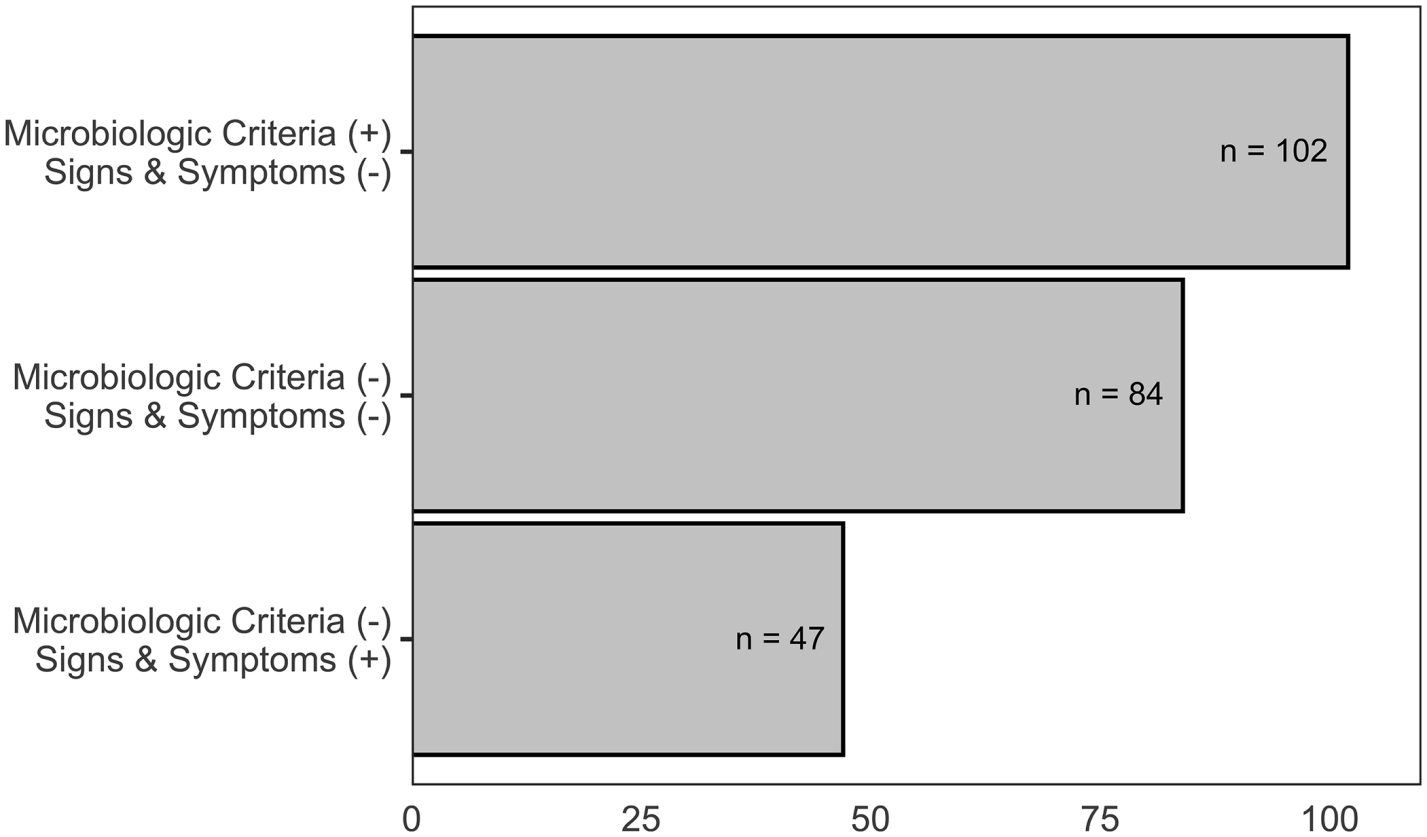
Classification of potential stop opportunities stratified by microbiologic criteria and presence of symptoms. Despite not meeting microbiologic or symptom criteria for urinary tract infections, 233 antibiotic events were continued. The 233 antibiotic events were stratified by urine culture results and symptom findings with the highest frequency (44%) occurring in asymptomatic bacteriuria: microbiologic criteria (+), signs and symptoms (−).

Of the 352 treatment courses assessed for a (stop PMO,” the continuation of 91 (25.9%) was justified based on the presence of suggestive resident symptoms and positive urine culture data. Another 28 treatment courses (8.0%) were stopped appropriately by the treating provider either due to negative urine culture results or lack of suggestive symptoms within 48 hours. The remaining 233treatment courses (66.2 %, totaling 1,900 days of therapy) were continued despite not meeting McGeer criteria or Loeb criteria with positive urine culture. Figure 1 shows the distribution of antibiotic treatments deemed to be amenable to a “stop PMO” stratified by the different combinations of clinical criteria and urine culture results. A plurality of these treatments (43.8%) was identified in residents with positive urine cultures but an absence of supportive signs or symptoms (asymptomatic bacteria). A significant proportion of antibiotic treatments were continued in residents with negative urine cultures as well as an absence of supportive signs or symptoms (36.0%). Residents who had supportive signs and symptoms but a negative urine culture were relatively infrequent (20.2%). Implementation of “stop PMO” at 72 hours identified 1,204 potentially avoidable antibiotic days of therapy (mean 5.2 days per nursing home resident).

### Shorten PMO

In total, 286 antibiotic courses were evaluated for a “shorten PMO.” The average microbiologically active antibiotic duration was 7.9 days (SD 2.8) with slightly longer courses prescribed for male compared to female residents (8.4 versus 7.6 days; *P* = .03). We detected no difference in average microbiologically active antibiotic duration between residents with or without an indwelling urinary catheter (*P* = .93). Of the 286 microbiologically active antibiotic treatments, 121 (42.3%) were prescribed for 7 days or fewer. The remaining 165 microbiologically active antibiotic treatment events (57.7%) were prescribed for >7 days and could have potentially been shortened, resulting in a total of 442 potentially avoidable days of antibiotic therapy, with a mean of 2.68 avoidable days per patient.

### Nonfluoroquinolone alternative PMO

A fluoroquinolone was prescribed for UTI in 145 (40.7%) of the eligible antibiotic courses. The 117 cases in which a fluoroquinolone was prescribed, and urine culture data were available for review were assessed for a nonfluoroquinolone alternative PMO. In 28 antibiotic courses (23.9%), fluoroquinolones were appropriately prescribed for an organism that was resistant to other oral antibiotic options. Providers changed to a nonfluoroquinolone alternative in a further 22 antibiotic courses (18.8%) after the urine culture data became available. Despite the availability of a nonfluoroquinolone alternative, 67 (57.3%) of the initially prescribed fluoroquinolone antibiotic courses were continued. Implementation of nonfluoroquinolone alternative PMO at 72 hours could remove up to 374 fluoroquinolone days of therapy.

After examining the eligible antibiotic treatment courses for each potential modification opportunity, only 59 (16.6%) were deemed to be not amenable to any modification. The remaining antibiotic courses (n = 297, 83.4%) were eligible for at least 1 PMO: 154 (43.3%) were eligible for 1 PMO, 118 (33.1%) were eligible for 2 PMOs, and 25 (7.0%) were eligible for all 3 PMOs (Table 3). Isolated “stop PMOs” only represented 33.3% of all PMOs, and 48.1% of antibiotics that could be modified were eligible for >1 PMO type. Coordinated implementation of all 3 PMOs at 72 hours could eliminate 1,326 avoidable antibiotic days of therapy and 32 avoidable fluoroquinolone days of therapy.

**Table 3.** Frequency of Potential Modification Opportunities (PMOs)

Opportunity	Antibiotic Events Eligible, No. (%)
Any PMO	297 (83.4)
**1 PMO**	154 (43.3)
Stop	99 (27.8)
Shorten	43 (12.1)
Non-FQ Alternative	12 (3.4)
**2 PMOs**	118 (33.1)
Stop & shorten	88 (24.7)
Stop & non-FQ alternative	21 (5.9)
Shorten & non-FQ alternative	9 (2.5)
**3 PMOs**	
Stop, shorten, & non-FQ alternative	25 (7.0)

Note. FQ, fluoroquinolone.

## Discussion

Our findings suggest that nearly 85% of antibiotic courses prescribed for treatment of a suspected UTI in nursing homes are potentially amenable to at least 1 type of postprescriptive modification. Discontinuation of initiated antibiotics was the most frequently identified postprescriptive modification opportunity identified in this study. Nearly 80% of these treatments were initiated in residents without significant localizing urinary signs or symptoms, and 56% of the residents continued to receive antibiotic treatment despite a subsequently negative urine culture (Fig. 1). Also, 57.5% of the antibiotic courses deemed to be amenable to discontinuation in this study were also amenable to modifications to shorten and/or change to a nonfluoroquinolone alternative, highlighting the potentially large impact of postprescriptive interventions on the quality of antibiotic prescribing in nursing homes.

The high frequency of treatment for apparent asymptomatic bacteriuria identified in the current study has been well described in other studies.^15,16^ The high prevalence of bacteriuria and impaired cognition among nursing home residents,^17^ as well as a persistent but unfounded belief that isolated behavioral symptoms are reliable indicators of UTI,^18,19^ are major factors contributing to this problem. Preprescriptive interventions that target the upstream decision to order a urine culture^20,21^ as well as interventions that promote limiting treatment to those residents manifesting specific signs and symptoms of UTI^22–24^ have recently been shown to reduce unnecessary antibiotic starts in nursing homes. Approximately 75% of the total antibiotic days from unnecessary treatment courses could be avoided through review and discontinuation 48 hours after treatment initiation, and ∼63% of total antibiotic days could be avoided if a review was conducted 72 hours after treatment initiation. These results suggest that layering a postprescriptive intervention on a preprescriptive intervention may be beneficial.

Clinicians may be more receptive to recommendations to modify rather than discontinue an antibiotic treatment course.^25^ Nearly 60% of the evaluable treatment courses in the current study exceeded 7 days despite accumulating evidence that most UTIs in older adults can be effectively treated with ≤7 days of antibiotics.^26,27^ Shortening these treatment regimens through a postprescriptive stewardship intervention would have avoided an additional 442 days of antibiotic exposure in our study cohort. Importantly, treatment guidelines available during the time study data were generated recommended longer treatment durations for men with UTI compared to women.^28^ Significant differences in UTI treatment length were observed between men and women in the current study. Consequently, our findings may overestimate the frequency of shortening modification opportunities in current times.

Fluoroquinolones were the most frequently prescribed antibiotic class for the treatment of suspected UTI in the current study (Table 1). Preprescriptive stewardship interventions have been successful in reducing the prescription of fluoroquinolone antibiotics in nursing homes and have further been associated with a reduced risk of CDI.^29^ Our results suggest that a significant number of potentially unnecessary fluoroquinolone days could be avoided through postprescriptive stewardship interventions. Whether a postprescriptive approach is of additive value in facilities with robust preprescriptive fluoroquinolone reduction programs requires additional study. The US Food and Drug Administration first added a “box warning” to fluoroquinolones in July 2008 and has subsequently amended this warning in 2013, 2016, and 2018.^30^ Consequently, we may have overestimated the frequency of fluoroquinolone prescribing in current times. However, contemporary studies have demonstrated that fluoroquinolones remain among the most frequently prescribed antibiotics for treatment of UTI in nursing homes.^3^

Postprescriptive stewardship interventions have primarily been evaluated in hospital settings, and the issues of how to best implement them and their effects on antibiotic prescribing practices in nursing homes remain understudied. Prospective audit with feedback (PAF) is the most employed and effective form of postprescriptive stewardship intervention in hospitals.^31,32^ Traditionally, hospital-based PAF programs are resource-intensive endeavors that are structured around infectious disease specialists and robust information systems,^33^ although lesser-resourced hospital-based PAF programs have also been effective.^34,35^ Although not structured as a classic PAF program, implementation of an infectious disease consultative service in a 160-bed Veterans’ Affairs nursing home that largely focused on postprescriptive modification of existing antimicrobial prescriptions resulted in 30.1% decrease in overall antibiotic use.^11^ Implementation of a weekly infectious pharmacist-led PAF program in 3 California nursing homes led to a 26% reduction in antibiotic prescribing for UTI.^12^ The antibiotic timeout is a distributed postprescriptive model that trains and supports frontline providers to modify existing antibiotic prescriptions based on diagnostic study results and the patient’s clinical trajectory.^36^ The effects of this form of self-stewardship intervention on prescribing patterns in the hospital setting have been mixed.^36,37^ Antibiotic timeouts are not widely used in nursing homes,^8^ and their effects on nursing home prescribing have been modest.^10,38^

This study had several limitations. First, the data used in the current study were collected using information available in resident health records. Important aspects of resident health status may have been omitted from these records, which could have led to overestimation of the number of modification opportunities identified in this study. Second, the explicit criteria employed during the assessment of the clinical appropriateness of antibiotic treatments^13,14^ are imperfect, which could have contributed to the overestimation of modification opportunities. Third, the current study was conducted using data on nursing home antibiotics that were prescribed in 2013 and 2014. Although national nursing home antibiotic prescribing patterns were stable from 2012 through 2016,^39^ i the 2016 release of regulations requiring nursing homes to implement antibiotic stewardship programs^40^ may have altered prescribing practices in ways that may limit the generalizability of our findings. Finally, our study is based on sample of only 5 nursing homes in Wisconsin, which may further limit the generalizability of our findings.

In summary, we identified a high frequency of postprescribing modification opportunities among antibiotic courses prescribed for the treatment of suspected UTI in a sample of Wisconsin nursing homes. Our study identified opportunities to discontinue, shorten, and modify these treatments, and only a minority of antibiotic prescriptions were not amenable to at least 1 modification opportunity. Although these results likely represent a best-case estimate of the amount of antibiotic modification achievable through postprescriptive interventions, even a moderate to large attenuation of findings would support a potential role for this stewardship approach in nursing homes. Additionally, our study excluded review of hospital-initiated UTI treatment courses, which may provide additional opportunities to enhance the quality of UTI antibiotic prescribing at the transition between hospitals and nursing homes. The actual returns achievable with postprescriptive interventions in the real world (whether they add incremental value when layered on preprescriptive stewardship interventions), their cost-effectiveness and issues related to their implementation in nursing homes require further study.
